# Impact of a Chicago Public Schools Network Specialist Supporting Health and Wellness Policy Implementation on Student Grades

**DOI:** 10.1111/josh.70106

**Published:** 2025-12-25

**Authors:** Julien Leider, Jeremiah Simon, Jamie Tully, Tarrah DeClemente, Elizabeth Jarpe‐Ratner, Jamie F. Chriqui

**Affiliations:** ^1^ Institute for Health Research and Policy, School of Public Health, University of Illinois Chicago Chicago Illinois USA; ^2^ Office of Student Health and Wellness, Chicago Public Schools Chicago Illinois USA; ^3^ Division of Health Policy and Administration, School of Public Health University of Illinois Chicago Chicago Illinois USA

**Keywords:** academic performance, health promotion, implementation, school health services, schools

## Abstract

**Background:**

School health and wellness‐related policies are associated with improvements in student health, but implementation varies. A Healthy Chicago Public Schools (CPS) Network Specialist position was developed to support policy implementation in one of 13 CPS elementary/middle school networks. This quasi‐experimental study examines the impact of a Specialist position on student academic performance.

**Methods:**

Self‐reported grades were obtained from 2021 to 2023 middle school Youth Risk Behavior Survey data (*N* = 3651). Grade point averages (GPAs) for Grade 6–8 students were obtained from administrative data for the 2018–2019 and 2022–2023 school years (*N* = 12,409). Difference‐in‐differences models were computed comparing changes in the network receiving Specialist supports and a comparison network.

**Results:**

The Specialist positively impacted self‐reported grades (adjusted odds ratio: 2.23; 95% confidence interval [CI]: 1.12–4.42), with the adjusted prevalence of students earning mostly As or Bs increasing in the intervention network (75.08%–78.22%) while declining in the comparison network (84.40%–74.47%). Student GPAs showed a marginal trend toward improvement (coefficient: 0.15; 95% CI: −0.01 to 0.31, *p* = 0.07).

**Implications:**

District‐level health and wellness policy implementation navigators can further schools' academic mission, supporting adoption of similar positions in other districts.

**Conclusions:**

Supports to schools to aid implementation of health and wellness‐related policies can benefit student academic performance.

Schools are subject to a multitude of federal, state, and district policies intended to improve student health, such as federal policies on school meals, state‐level wellness‐related policies, and district‐level local wellness policies [1]. Studies have shown associations between such policies and improvements in student health outcomes including greater physical activity, better dietary intake, and improved outcomes from health screenings and preventive care [2, 3, 4, 5]. Further, improved student health is associated with better academic performance [6, 7, 8, 9, 10]. However, policy implementation by schools varies and is subject to a number of barriers, including limited funding, lack of time and competing priorities, and lack of supports for implementation [11, 12, 13, 14, 15]. These barriers have been heightened by the ongoing impacts of school closures in response to the COVID‐19 pandemic and ensuing disruptions to schools and the students they serve, including student learning loss and mental health challenges [16, 17].

Chicago Public Schools (CPS) is the fourth largest school district in the United States [18] with a diverse student body [19] and a majority of students from families with income ≤ 185% of the federal poverty line [20]. CPS launched the Healthy CPS initiative in 2016 to promote compliance with more than 50 federal, state, and district health and wellness‐related policies [21]. As part of this initiative, schools are rated annually on the extent to which they have implemented policies in four “badge” areas: Health Leadership, Health Instruction, Healthy Environments, and Health Services. Schools that achieve at least 90% compliance are considered to have achieved the given badge, and this is noted in public reports [22].

In 2019, CPS began working in partnership with the Policy, Practice, and Prevention Research Center (P3RC) at the University of Illinois Chicago (UIC) School of Public Health on a research project to support Healthy CPS compliance in one CPS network on the West Side of Chicago, an area of the city that has faced longstanding disinvestment and inequity and whose residents are primarily people of color. This was the core research project for the UIC P3RC, one of 26 Centers for Disease Control and Prevention‐funded Prevention Research Centers between 2019 and 2024. The selected network was one of 13 into which CPS elementary and middle schools are divided; each network is akin to a “mini‐district” and is similar in size to a typical school district [23]. A Healthy CPS Network Specialist position was developed collaboratively by CPS and researchers at UIC to provide technical assistance and serve as a navigator to the elementary and middle schools in this network. The underlying premise for the Network Specialist position was based upon the literature noted above; if schools were successful in implementing health and wellness policies (by working with a Network Specialist), students would be healthier overall, and the above evidence indicates that healthier students have better academic outcomes.

The Network Specialist was hired and began supporting schools in 2020, as school closures in response to the COVID‐19 pandemic began. They worked with schools remotely in school year 2020–2021, on a hybrid schedule in school year 2021–2022, and fully in‐person beginning in school year 2022–2023.

The Network Specialist position was created to provide a single point of contact to support schools in the one intervention network in all aspects of implementing Healthy CPS. The Specialist met regularly with each school throughout the school year to review their current performance as measured by their Healthy CPS score (computed annually based on survey and district administrative data) and develop and implement action plans to achieve school health and wellness‐related priorities [21]. As detailed in previous work, one of the key roles of the Specialist was to connect schools to relevant people and resources both within and outside the district [24]. The Specialist provided supports using a Multi‐Tiered System of Supports framework in which schools receive more or less intensive supports based on their level of need, ranging from Tier 1 supports provided universally to all schools (e.g., general informational emails) to Tier 2 (e.g., phone call to explain sexual health education requirements) and Tier 3 supports (e.g., meeting with a school to develop a detailed Healthy CPS implementation plan) [21, 25, 26, 27]. Table 1 provides further examples of the types of supports offered by tier.

**TABLE 1:** Examples of tiers of support provided by the Healthy CPS Network Specialist.

Tier of support	Example supports provided
Tier 1 (core supports)	Introductory email with updates on Healthy CPS Reminder email to assign a Medicaid designee Email with information about nutrition‐related policies and nutrition education resources
Tier 2 (supplementary supports)	Call to explain sexual health education requirements Call to discuss staff training rates Call to connect school with external partner to support school garden
Tier 3 (individualized, intensive supports)	Meeting with a school to develop a detailed Healthy CPS implementation plan Meeting to discuss strategies to improve School Medical Form completion rates

The current study examined the impact of the Network Specialist on academic performance using a quasi‐experimental design. Considering the literature linking improved student health with better academic performance, it was hypothesized that the Network Specialist supports for health and wellness policy implementation would lead to improvements in student academic outcomes in the Specialist's network as compared to a sociodemographically matched comparison network. One of CPS' goals with the project was to provide evidence for district leadership on the viability of this type of role, given schools' and districts' focus on their primary educational mission. To the authors' knowledge, only two previous studies have examined this type of school health navigator role [24, 28], and neither examined impacts on academic outcomes. This study seeks to fill that gap by specifically examining the impact of a navigator on improving student academic outcomes, which is necessary for school districts, such as CPS, to justify the value of such a position to district leadership.

## 1 Methods

### 1.1 Study Design and Data Sources

1.1.1

This study employed a pre–post, intervention‐comparison site design in which changes in performance in the intervention network, which received Specialist supports, were compared to changes in performance in a comparison network that only received standard district‐level supports (e.g., universal supports and information dissemination, nothing tailored, or customized to the individual school).

Repeated cross‐sectional data on student‐level academic performance and student‐level characteristics were obtained from two datasets. First, CPS middle school YRBS data were collected in Fall 2021 and Spring 2023 using standard YRBS methodology [29, 30]. This study relied on unique YRBS subsite data collected separately for the intervention and comparison networks. Second, administrative data on middle school student grade point averages (GPAs) in the intervention and comparison networks were obtained from the district for the 2018–2019 and 2022–2023 school years.

### 1.2 Participants

#### 1.2.1 YRBS Participants

A total of 4194 valid responses to the middle school YRBS were obtained across the intervention and comparison networks and both data collection years. These were special subsite YRBS data collection efforts that were designed to be representative of each network. Complete case analysis was used, so responses were excluded from the analysis where they were missing data on student characteristics including race and ethnicity (87 responses), sex (23), and grade level (3), or where they were missing data on the outcome of self‐reported grades (430 responses). The final analytical sample included 3651 student responses over the 2‐year period.

#### 1.2.2 GPA Data From Students

Administrative GPA data were obtained for a total of 13,117 observations across networks and school years. Because of 708 observations with missing data on the GPA outcome, the final analytical sample included 12,409 student‐level observations.

### 1.3 Instrumentation

#### 1.3.1 YRBS Data

The outcome of interest was computed from a question asking students, “during the past 12 months, how would you describe your grades in school?” Response options included mostly As, mostly Bs, mostly Cs, mostly Ds, mostly Fs, none of these grades, and not sure. Analyses examined a dichotomous measure of students reporting earning mostly As or Bs versus Cs, Ds, or Fs, treating responses of “none of these grades” or “not sure” as missing.

Student race and ethnicity, sex, and grade level were also obtained from the surveys. Student race and ethnicity were self‐reported based on questions asking “Are you Hispanic or Latino?” (response options of yes or no) and “What is your race? (Select one or more responses)” (response options of “American Indian or Alaska Native,” “Asian,” “Black or African American,” “Native Hawaiian or Other Pacific Islander,” and “White”). For analyses, a combined measure of race and ethnicity was computed with categories of Black or African American, Hispanic/Latinx, White, and all other races (the latter had to be combined due to small sample sizes for other and multiple race selections). Student sex was obtained from a question asking “What is your sex?” with response options of “female” and “male.” Grade level was obtained from a question asking “In what grade are you?” with response options of “6th grade,” “7th grade,” “8th grade,” and “ungraded or other grade.”

#### 1.3.2 GPA Data

Final quarter four GPAs of grade 6–8 students in the intervention and comparison networks as of school years 2018–2019 and 2022–2023, linked to student characteristics, were obtained from district administrative data. Student characteristics included race and ethnicity, gender (female, male, or non‐binary), grade level, and indicators for: eligible for free/reduced‐price meals, homeless, English as a second language, having an Individualized Education Program (IEP), and having a 504 plan. Student‐level race and ethnicity were categorized as American Indian, Asian, Black, Hawaiian or Pacific Islander, Hispanic, Multi, N/A, and White. Because of small sample sizes, race and ethnicity were collapsed as Black, Hispanic, White, and all other races.

### 1.4 Data Analysis

A difference‐in‐differences analytic approach was used to investigate the causal impact of the Network Specialist on student academic outcomes. The resulting estimates are causal under the parallel trends assumption, that is, that changes in the outcome would have been the same in both the intervention and comparison networks in the absence of the Specialist intervention [31]. While this assumption is not testable, the comparison network was chosen to be sociodemographically comparable and was also on the West Side, supporting its plausibility.

Using the YRBS data, a logistic regression DID model was computed to assess the impact of the Network Specialist intervention on students describing their grades as mostly As or Bs, adjusting for the student characteristics noted above and accounting for the YRBS survey design and weights. Using the administrative GPA data, a linear regression DID model was computed to assess the impact of the intervention on student GPAs, adjusting for student characteristics and with robust standard errors clustered on school. Analyses did not account for clustering on network because only two networks were examined. Adjusted prevalence estimates were computed from the YRBS model and adjusted mean estimates were computed from the GPA model. Analyses were conducted in Stata/MP 18.0.

## 2 Results

Tables 2 and 3 show characteristics of the analytical samples for analyses of the YRBS and GPA data. The unadjusted prevalence of students reporting earning mostly As or Bs increased in the intervention network from 73.64% to 76.92% while decreasing in the comparison network from 85.18% to 75.37%. Similarly, the unadjusted mean GPA increased from 2.65 to 2.79 in the intervention network while declining slightly from 2.90 to 2.87 in the comparison network. Both intervention network samples included about two‐thirds of students identifying as Black and one‐third identifying as Hispanic, while this was reversed in the comparison network samples with two‐thirds of students identifying as Hispanic and one‐third identifying as Black. Both samples were roughly evenly split across grade levels 6–8 and by sex (YRBS) or gender (GPA data). Characteristics from the GPA data showed the overwhelming majority of students (89%–94%) were eligible for free or reduced‐price meals, about one‐fifth (19%–22%) had an Individualized Education Program, 9%–32% spoke English as a second language, and fewer than 10% had a 504 plan or were homeless.

**TABLE 2:** Characteristics of analytical sample of Chicago Public Schools Middle School Youth Risk Behavior Survey (YRBS) respondents.^a^

	Intervention network		Comparison network	
	Fall 2021	Spring 2023	Fall 2021	Spring 2023
Outcome				
Described grades as mostly As or Bs	73.64%	76.92%	85.18%	75.37%
Student characteristics				
Race and ethnicity				
Black or African American	63.59%	63.02%	30.59%	34.27%
Hispanic/Latinx	31.87%	34.95%	65.25%	61.69%
White	2.14%	0.42%	1.96%	2.00%
All other races	2.39%	1.61%	2.20%	2.05%
Sex				
Female	46.53%	48.53%	50.53%	50.95%
Male	53.47%	51.47%	49.47%	49.05%
Grade level				
6th grade	28.97%	31.88%	32.96%	29.75%
7th grade	32.70%	33.76%	30.48%	34.23%
8th grade	38.33%	34.10%	36.56%	35.79%
Ungraded or other grade	0.00%	0.25%	0.00%	0.23%

^a^ *N* = 3651 responses from 711 students in the intervention network and 884 students in the comparison network in Fall 2021, and from 874 students in the intervention network and 1182 students in the comparison network in Spring 2023. Statistics were computed taking into account the YRBS survey design and weights.

**TABLE 3:** Characteristics of analytical sample of Chicago Public Schools Middle School Students with Administrative Data on Grade Point Averages.^a^

	Intervention network		Comparison network	
	School year 2018–2019	School year 2022–2023	School year 2018–2019	School year 2022–2023
Outcome (mean [SD])				
Grade point average	2.65 (0.82)	2.79 (0.67)	2.90 (0.78)	2.87 (0.74)
Student characteristics (%)				
Race and ethnicity				
Black	65.42%	63.73%	34.24%	31.70%
Hispanic	32.15%	33.03%	62.04%	64.45%
White	1.45%	2.16%	2.42%	2.42%
All other races	0.98%	1.08%	1.30%	1.44%
Gender				
Female	50.68%	47.30%	49.85%	48.40%
Male	49.23%	52.64%	50.13%	51.60%
Non‐binary	0.09%	0.06%	0.02%	0.00%
Grade level				
6th grade	37.67%	33.03%	37.52%	33.94%
7th grade	31.82%	31.38%	31.43%	32.91%
8th grade	30.51%	35.59%	31.05%	33.16%
Eligible for free/reduced‐price meals	94.34%	91.47%	92.19%	88.69%
Homeless	9.12%	9.27%	3.81%	7.81%
English is second language	8.52%	14.44%	16.40%	32.17%
Have an IEP	21.81%	20.35%	19.61%	18.80%
Have a 504 plan	4.73%	6.25%	4.60%	5.72%

Abbreviation: IEP, Individualized Education Program.

^a^ *N* = 12,409 observations, including 2137 students in the intervention network and 4544 students in the comparison network as of the 2018–2019 school year, and 1759 students in the intervention network and 3969 students in the comparison network as of the 2022–2023 school year.

**FIGURE 1:**
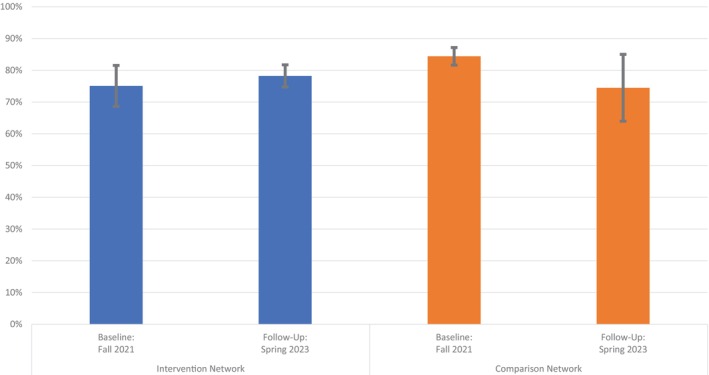
Changes in the prevalence of students reporting earning mostly As and Bs, Youth Risk Behavior Survey (YRBS), Fall 2021–Spring 2023. The adjusted prevalence of students reporting earning mostly As or Bs from logistic regression difference‐in‐differences models adjusting for student race and ethnicity, sex, and grade level and accounting for the YRBS survey design and weights is shown. Error bars correspond to 95% confidence intervals around the estimates. The difference between the changes in the intervention and comparison networks was statistically significant (adjusted odds ratio: 2.23; 95% confidence interval: 1.12–4.42, *p* = 0.02), indicating the intervention had a positive impact on student self‐reported grades.

Figure 1 shows the results of the DID model examining the impact of the Network Specialist intervention on student self‐reported grades from the YRBS data. From Fall 2021 to Spring 2023, the adjusted prevalence of middle school students earning mostly As or Bs increased from 75.08% to 78.22% in the intervention network, while declining from 84.40% to 74.47% in the comparison network. The difference between the changes in the intervention and comparison networks was statistically significant (adjusted odds ratio: 2.23; 95% confidence interval: 1.12–4.42, *p* = 0.02; not shown in tables or figures), indicating the intervention had a positive impact on student self‐reported grades.

**FIGURE 2:**
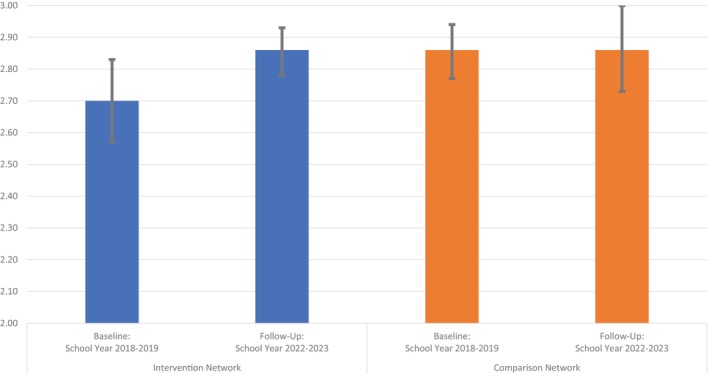
Changes in student grade point averages (GPAs), school years 2018–2019 to 2022–2023. Adjusted mean GPAs are shown from linear regression difference‐in‐differences models with robust standard errors clustered on school adjusting for the student characteristics in Table 3. Error bars correspond to 95% confidence intervals around the estimates. The difference between the changes in the intervention and comparison networks was marginally significant (coefficient: 0.15; 95% confidence interval: −0.01, 0.31, *p* = 0.07), suggesting the intervention had a positive impact on student GPAs.

Figure 2 shows the results of DID models examining student GPAs using administrative data. The Network Specialist intervention was marginally associated with an increase in average GPAs in the intervention relative to the comparison network. From school year 2018–2019 to 2022–2023, adjusted mean GPAs went from 2.70 to 2.86 in the intervention network while remaining stable at 2.86 in the comparison network. The DID estimate showed a marginal trend toward improvement (coefficient: 0.15; 95% confidence interval: −0.01, 0.31, *p* = 0.07; not shown in tables or figures)

## 3 Discussion

This study found that receiving technical assistance from a Network Specialist to support the implementation of school health and wellness‐related policies was associated with improvements in academic performance relative to a comparison network. While no previous study has examined the association of this type of position with academic performance, this is consistent with previous research showing associations between school health and wellness‐related policies and student health outcomes [2, 3, 4, 5] and the documented association between improved student health and better academic performance [6, 7, 8, 9, 10].

Previous research has highlighted that one of the key roles filled by this type of position is to help connect schools to resources and people, both inside and outside the school system, that can help schools meet policy requirements [24, 28]. The Network Specialist worked to improve school practices and, in turn, student‐level outcomes by facilitating and supporting the implementation of school health and wellness‐related policies and practices, such as by connecting a school with an external partner to help with their school garden, connecting schools that had already had success in implementation with others that could learn from them, or meeting with a school to develop a detailed implementation plan [21, 24]. Lack of time and competing priorities constitute one of the main barriers to policy implementation [11, 12, 13, 14, 15], and in this environment having a single point of contact can be a crucial support in connecting schools with the resources they need [13]. In the context of a different but similarly complex system, patient navigators have helped those suffering from chronic diseases to adhere to treatment guidelines by helping them navigate the healthcare system, connecting them with community resources, and educating them about disease [32, 33]. This study suggests that a similar approach to helping schools navigate the numerous health and wellness‐related policies they are subject to can improve student academic performance.

This study found a statistically and practically significant relative increase in the percentage of students self‐reporting earning mostly As and Bs, as this increased by several percentage points in the intervention network while falling by 10 percentage points in the comparison network. It also found a marginal trend toward improvement in student GPAs, with a marginally significant increase of 0.15 in the mean GPA in the intervention network relative to the comparison network following the Network Specialist intervention. While this is not a large effect in absolute terms, it represents a 6% increase relative to the baseline adjusted mean GPA in the intervention network. This is still meaningful, particularly when considering students faced significant inequities, with schools located in an area of the city subject to historical disinvestment and more than 90% of students eligible for free or reduced‐price meals, and the fact that higher middle school GPAs are associated with higher GPAs in high school [34].

This study was conducted in the midst of the COVID‐19 pandemic. The Network Specialist began their work in 2020 as school closures began to limit the spread of COVID‐19, and they were forced to work remotely or on a hybrid schedule during their first two school years. Prior work has documented significant learning losses following the pandemic, particularly for disadvantaged students, as well as disruptions to school environments and deteriorations in student mental health [16, 17, 35, 36, 37]. The baseline YRBS data for this study were collected in Fall 2021 just as Chicago Public Schools moved back to fully in‐person learning [38], and the baseline GPA data were collected prior to the pandemic. The findings of improvements in academic performance associated with the Network Specialist intervention are particularly remarkable in this context.

### 3.1 Implications for School Health Policy, Practice, and Equity

CPS has been expanding Network Specialist supports to other CPS networks. Based on this study's results highlighting the potential of this type of position to enhance student academic performance, which in turn can lead to continued benefits later in their schooling [34], other districts should consider providing similar supports. While CPS is the fourth largest school district in the United States [18], this position was limited to one network with fewer than 10,000 students, so findings are applicable both to other large school districts with a similar network structure as well as small‐ to medium‐sized districts that could apply such a position to their entire district. Other districts seeking to provide similar supports can adapt resources that have already been developed as part of the current Network Specialist project [21, 25, 39].

### 3.2 Limitations

While this study makes a significant contribution as the first to examine the impact of this type of technical assistance on student academic performance and employs a strong research design including a comparison network to account for secular changes within the district, it is nonetheless subject to several limitations. First, the parallel trends assumption on which the difference‐in‐differences analyses rely is not testable, and we did not have data on multiple pre‐intervention timepoints to provide evidence on its plausibility, although the choice of a comparison network from the same district and area of the city lends it credibility. Second, while the comparison network was selected to be sociodemographically comparable to the intervention network, there were some differences in student characteristics, particularly with students in the intervention network being mostly Black and students in the comparison network being mostly Hispanic, as shown in Tables 2 and 3. Third, baseline YRBS data were collected in Fall 2021, after the Specialist intervention had already been in place for more than a year; this delayed fielding was an artifact of the COVID‐19 pandemic. As a result, analyses of the YRBS data may understate the impact of the intervention by failing to incorporate improvements that had already taken place at that point. Fourth, the YRBS data on grades are self‐reported and thus subject to reporting error. However, the analyses relying on district administrative data on GPAs yielded similar results, increasing confidence in the study's findings. Finally, this study examined two elementary and middle school networks that have faced historical disinvestment and that are part of a large urban school district, and results may not be generalizable to other settings.

## 4 Conclusions

This study shows the value the Healthy CPS Network Specialist provided in terms of enhanced student academic performance as a result of the supports they provided to schools in implementing health and wellness‐related policies. Other districts should consider creating similar positions, and future research should further explore how this type of position can best support schools using both quantitative and qualitative methods.

## Funding

This work was supported by the Centers for Disease Control and Prevention (6 NU87PS004311, U48DP006392).

## Ethics Statement

This study was reviewed and approved by the University of Illinois Chicago Institutional Review Board (protocol 2019‐1161).

## Conflicts of Interest

The authors declare no conflicts of interest.

## Data Availability

The YRBS data that support the findings of this study are available on request from the corresponding author. The data are not publicly available due to privacy or ethical restrictions. The GPA data are not available for sharing as they were obtained by the authors under a restricted use agreement with CPS and contain identifying information.
